# Increased risk of virological failure to the first antiretroviral regimen in HIV-infected migrants compared to natives: data from the ICONA cohort

**DOI:** 10.7448/IAS.17.4.19769

**Published:** 2014-11-02

**Authors:** Annalisa Saracino, Patrizia Lorenzini, Sergio Lo Caputo, Enrico Girardi, Francesco Castelli, Paolo Bonfanti, Massimo Galli, Pietro Caramello, Nicola Abrescia, Cristina Mussini, Laura Monno, Antonella d'Arminio Monforte

**Affiliations:** 1 Clinic of Infectious Diseases, University of Bari, Bari, Italy; 2 Clinical Department, National Institute for Infectious Diseases “L. Spallanzani” IRCCS, Rome, Italy; 3 Clinic of Infectious Diseases, Santissima Annunziata Hospital, Florence, Italy; 4 Department of Epidemiology, National Institute for Infectious Diseases “L. Spallanzani’’ IRCCS, Rome, Italy; 5 Division of Infectious and Tropical Diseases, Civili General Hospital, University of Brescia and Spedali Brescia, Italy; 6 Department of Infectious Diseases, Azienda Ospedaliera Lecco, Lecco, Italy; 7 Department of Infectious Diseases, L. Sacco University Hospital, University of Milan, Milan, Italy; 8 Department of Infectious Diseases, Amedeo di Savoia Hospital, Turin, Italy; 9 Department of Infectious Diseases, Cotugno Hospital, Naples, Italy; 0 Clinic of Infectious Diseases, University of Modena and Reggio Emilia, Modena, Italy; 1 Clinic of Infectious Diseases, San Paolo Hospital, University of Milan, Milan, Italy

## Abstract

**Introduction:**

Aim of the study was to evaluate possible disparities in access and/or risk of virological failure (VF) to the first antiretroviral (ART) regimen for migrants compared to Italian-born patients and to assess determinants of failure for the migrants living with HIV.

**Methods:**

All native and migrant naïve patients enrolled in ICONA in 2004–2014 were included. Firstly, variables associated to ART initiation were analyzed. In a second analysis, the primary endpoint was time to failure after at least six months of ART, defined as: (a) VF (first of two consecutive viral load (VL) >50 and >200 copies/mL); (b) treatment discontinuation (TD) for any reason; and (c) treatment failure (TF: confirmed VL >200 cp/mL or TD). A Poisson multivariable analysis was performed to control for confounders.

**Results:**

A total of 5777 HIV-pos ART-naïve patients (1179 migrants and 4598 natives) were evaluated. Most migrants were from sub-Saharan Africa (35.3%) and South-Central America/Caribbean (29%). Median duration of residency in Italy was five years (IQR 1–10). Baseline characteristics significantly differed between the two groups (Table 1); in particular, lower CD4 counts and higher frequency of AIDS events were observed in migrants vs natives. When adjusting for baseline confounders, migrants presented a lower chance to initiate ART compared to natives (OR 0.78, 95% CI 0.65–0.93, p=0.006). After ART initiation, the incidence rate of VF >50 cp/mL was 15.5 per 100 person-years (95% CI 12.8–18.8) in migrants and 8.9 in natives (95% CI 7.9–9.9), respectively. By multivariable analysis, migrants had a significantly higher risk of VF, both >50 cp/mL (OR 1.50, 95% CI 1.17–1.193, p=0.001) and >200 cp/mL (OR 1.59, 95% CI 1.23–2.05, p<0.001), and of TF (OR 1.15, 95% CI 1.00–1.32, p=0.045), while no differences were observed in TD risk. Among migrants, variables associated with a higher VF risk were age (for 10-year increase, OR 0.96, 95% CI 0.93–0.98, p=0.002), unemployment (OR 1.96, 95% CI 1.20–3.20, p=0.007) and use of a boosted PI based-regimen (OR 2.04, 95% CI 1.25–3.34, p=0.005 vs NNRTI-based), while pregnancy was associated with TD (OR 3.73, 95% CI 2.36–5.90, p<0.001) and TF (OR 3.13, 95% CI 02.00–4.89, p<0.001).

**Conclusions:**

Despite the use of more potent and safer antiretroviral drugs in the last 10 years, and even in a setting of universal access to ART, migrants living with HIV still present barriers to ART initiation and increased risk of VF compared to natives.

**Table 1:** Characteristics of migrant and native-born HIV-positive antiretroviral naïve patients enrolled in the ICONA cohort during the period 2004–2014 at time of inclusion

		Migrants	Natives	p
Total n=5777		n=1179	n=4598	
Male gender, n (%)		674 (57.2%)	3914 (85.1%)	<0.001
Age, yrs, median (IQR)		34 (28–40)	39 (32–47)	<0.001
Education, n (%)	Less than high	355 (30.1%)	1075 (17.3%)	<0.001
	High	309 (26.2%)	1980 (43.1%)	
	Missing	515 (43.7%)	1543 (33.6%)	
Employment, n (%)	Full employed	444 (37.7)	2786 (60.5%)	<0.001
	Occasionally employed	99 (8.4%)	78 (1.7%)	
	Unemployed	353 (30.0%)	554 (14.5%)	
	Other/missing	229 (19.4%)	1180 (25.7%)	
Mode of HIV transmission, n (%)	Heterosexual	703 (59.6%)	1634 (35.5%)	<0.001
	Homosexual	312 (26.5%)	2194 (47.7%)	
	IVDU	48 (4.1%)	436 (9.5%)	
	Other/unknown	116 (9.8%)	334 (7.3%)	
Recent drug use, n (%)	No	904 (76.7%)	3352 (72.9%)	0.005
	Yes	21 (1.8%)	149 (3.2%)	
	Unknown	254 (21.5%)	1097 (23.9%)	
Smoke, n (%)	No	677 (57.4%)	1757 (38.2%)	<0.001
	Yes	252 (21.4%)	1780 (38.7%)	
	Unknown	250 (21.2%)	1061 (23.1%)	
Pregnancy status, n (%)		51 (4.3%)	17 (0.4%)	<0.001
HIV subtype, n (%)	B	203 (17.2%)	1344 (29.2%)	<0.001
	Non-B	218 (18.5%)	316 (6.9%)	
	Unknown	758 (64.3%)	2938 (63.9%)	
First HIV RNA (per 1 log cp/mL more)		4.5 (IQR 3.7–5.2)	1.6 (IQR 3.9–5.2)	0.066
First CD4 count (per 100 cell/mmc more)		317 (IQR 137–509)	396 (223–577)	<0.001
First CD4, cell/mmc	<200	324 (27.5%)	876 (19.1%)	0.003
	200–350	226 (19.2%)	732 (15.9%)	
	>350	440 (37.3%)	2197 (47.8%)	
	Missing	189 (16.0%)	793 (17.3%)	
AIDS event pre-treatment, n (%)		140 (11.9%)	362 (7.9%)	<0.001
HCV co-infection, n (%)	Positive	71 (6.0%)	446 (9.7%)	<0.001
	Negative	794 (67.3%)	2882 (62.7%)	
	Unknown	314 (26.6%)	1270 (27.6%)	
HBV co-infection, n (%)	Positive	58 (4.9%)	144 (3.1%)	0.008
	Negative	785 (66.6%)	3060 (66.6%)	
	Unknown	336 (28.5%)	1394 (30.3%)	
CMV co-infection, n (%)	Positive	45 (3.8%)	246 (5.3%)	<0.001
	Negative	492 (41.7%)	1579 (34.3%)	
	Unknown	642 (54.4%)	2773 (60.3%)	

